# Cardiovascular Effects of a Thoracoamniotic Shunt in a Fetus Affected by Isolated Right Congenital Diaphragmatic Hernia and Hydrops

**DOI:** 10.7759/cureus.54279

**Published:** 2024-02-16

**Authors:** Silvio Tartaglia, Carmela Paciullo, Daniela Visconti, Antonio Lanzone, Marco De Santis

**Affiliations:** 1 Department of Women's and Children's Health Sciences and Public Health, Fondazione Policlinico Universitario Agostino Gemelli Istituto Di Ricovero e Cura a Carattere Scientifico (IRCSS), Rome, ITA; 2 Department of Gynecology and Obstetrics, Università Cattolica del Sacro Cuore, Rome, ITA

**Keywords:** fetal cardiac function, combined cardiac output, thoracoamniotic shunt, fetal hydrops, congenital diaphragmatic hernia

## Abstract

A thoracoamniotic shunt was placed in a fetus affected by a right congenital diaphragmatic hernia (RCDH) complicated by voluminous nonimmune hydrops (NIH) at 30 weeks of gestation. The fetus showed congestive cardiac failure with a combined cardiac output (CCO) of 460.7 ml/min (Z-score: -1.2). After seven days, no edema, ascites, or pleural effusion was present. CCO increased significantly, reaching a Z-score of -0.2, as well as right and left cardiac output (Z-scores: -0.3 and -0.8, respectively). Two weeks later, the cardiac function and the ascites got worse despite the correct shunt placement, suggesting a possible occlusion. At 33 weeks, a C-section was performed due to labor in breech presentation. Despite the intensive care provided, the newborn died due to pulmonary hypertension and respiratory insufficiency. The thoracoamniotic shunt’s effect on fetal circulation and the mechanisms of NIH in the event of RCDH are still unclear. Due to the high mortality rate of this condition and its poorer outcomes compared to left-sided defects, shunting cannot be considered an efficient attempt to improve fetal and neonatal survival rates to date. A close relationship between the amount of lymphatic effacement and cardiac function is clear, but further studies are needed to provide more information about this severe condition and its treatment.

## Introduction

Congenital diaphragmatic hernia is a condition occurring in one in every 2000 to 4000 pregnancies, with right-sided cases, also known as right congenital diaphragmatic hernia (RCDH), representing only 10% to 15% [1]. One complication associated with RCDH is hydrops, a condition marked by abnormal accumulation of fluid in multiple fetal compartments, including the skin, abdomen, and thoracic cavity. Hydrops in the context of RCDH can further complicate the management and prognosis of the affected fetus, as they reflect an imbalance in fluid dynamics and can lead to compromised organ function. The suggested cause is caval compression, leading to decreased preload and impaired cardiac output [2]. The management of hydrops in cases of RCDH remains unclear. Thoracoamniotic shunt placement has emerged as a therapeutic strategy for treating fetal hydrothorax and hydrops [3]. This procedure involves the transamniotic insertion of a catheter into the fetal chest to create a connection between the thoracic and amniotic cavities, facilitating the drainage of excess fluid and potentially alleviating the pressure on the heart and the developing lungs. Understanding the intricacies of RCHD, hydrops, and the role of thoracoamniotic shunts is crucial for healthcare professionals involved in the care and decision-making processes for affected pregnancies.

In this discussion, we describe the longitudinal modification of the fetal cardiac output after thoracoamniotic shunt placement, evaluated by seriate ultrasound measuring combined cardiac output (CCO) as suggested by Mielke and Benda [4]. The aim is to better understand the effects of hydrops and their resolution on fetal heart function.

## Case presentation

**Figure 1:**
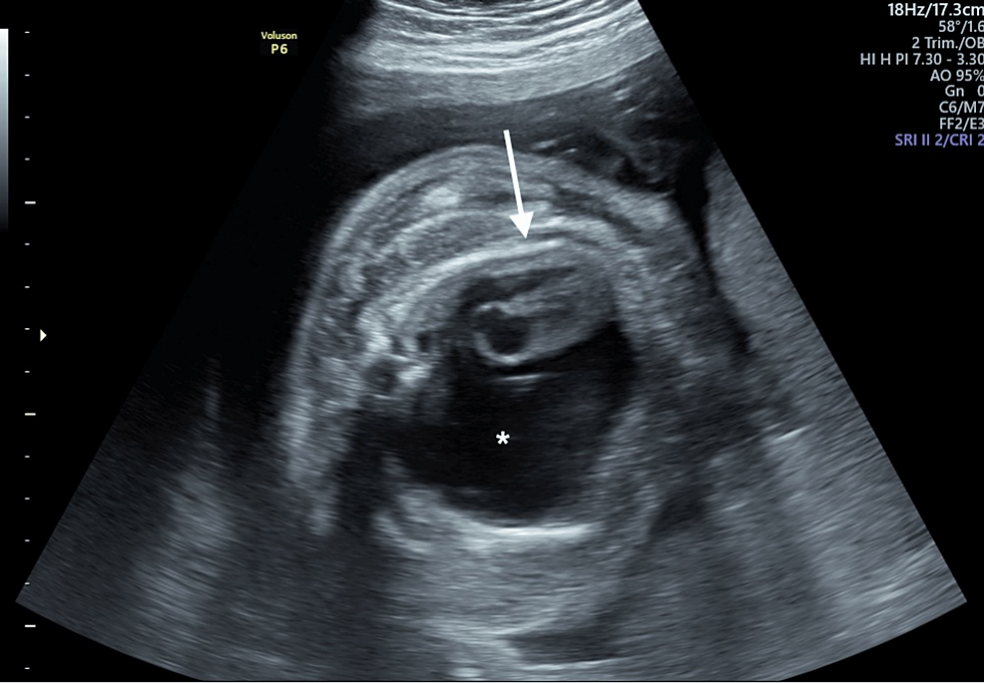
Axial plane of the fetal chest showing a massive right pleural hydrothorax (asterisk) with a mediastinal shift to the left (arrow)

A 35-year-old primigravida was referred to our institution during the third trimester for the recent onset of nonimmune hydrops (NIH). The second-trimester anomaly scan was reported as normal. At 29 weeks of gestation, the ultrasound revealed severe hydrops with subcutaneous evident edema, ascites, hydrocele, hydrothorax, and polyhydramnios. A voluminous right pleural effusion with a mediastinal shift to the left was present with no visualization of the right lung (Figure 1).

The longitudinal view of the right hemithorax revealed a “mushroom-like” liver herniation, suggesting an RCDH diagnosis with severe right lung hypoplasia confirmed at magnetic resonance. Fetal cardiac function, assessed by CCO, resulted lower than expected (460.7 ml/min, Z-score: -1.2 for gestational age, according to Rocha et al. [5]), and so was the right and left ventricle cardiac output. After multidisciplinary counseling, the couple gave consent to placing a right thoracoamniotic shunt. At 30 weeks of gestation, a 35-mm Harrison fetal bladder stent (Cook Medical Inc., Bloomington, IN, USA) was placed under continuous ultrasound guidance after the administration of tocolytics and intravenous anesthetic drugs to the mother. The procedure was uncomplicated. Ultrasonographic follow-up was performed the day after the procedure and then weekly (Table 1).

**Table 1:** Evolution of the hydrops features and progressive changes in CCO after thoracoamniotic shunt positioning CO: cardiac output, CCO: combined cardiac output, *according to Rocha et al. (2019) [5]

Ultrasound longitudinal evaluations	Before intervention	1 day after	7 days after	14 days after	21 days after
Perihepatic ascites (mm) (± %)	24	17 (-29.2%)	1 (-94.1%)	0 (-100%)	12 (+1200%)
Frontal edema (mm) (± %)	13.6	10 (-26.5%)	6 (-40%)	1 (-83.3%)	5 (+500%)
Hydrothorax (mm) (± %)	18	7 (-61.1%)	2 (-71.4%)	0 (-100%)	10 (+1000%)
Amniotic fluid, max vertical pocket (mm) (± %)	99	90 (-9.1%)	86 (-4.4%)	80 (-6.8%)	92 (+15%)
Left CO (ml/min) (Z-score*)	178.9 (-1.2)	161.3 (-1.3)	156.7 (-1.4)	218.8 (-0.8)	130.1 (-2.2)
Right CO (ml/min) (Z-score*)	281.8 (-1.2)	253.0 (-1.4)	283.3 (-1.2)	468.9 (-0.3)	382.6 (-1.0)
CCO (ml/min) (Z-score*)	460.7 (-1.2)	414.3 (-1.4)	409.7 (-1.4)	687.7 (-0.2)	512.7 (-1.4)

**Figure 2:**
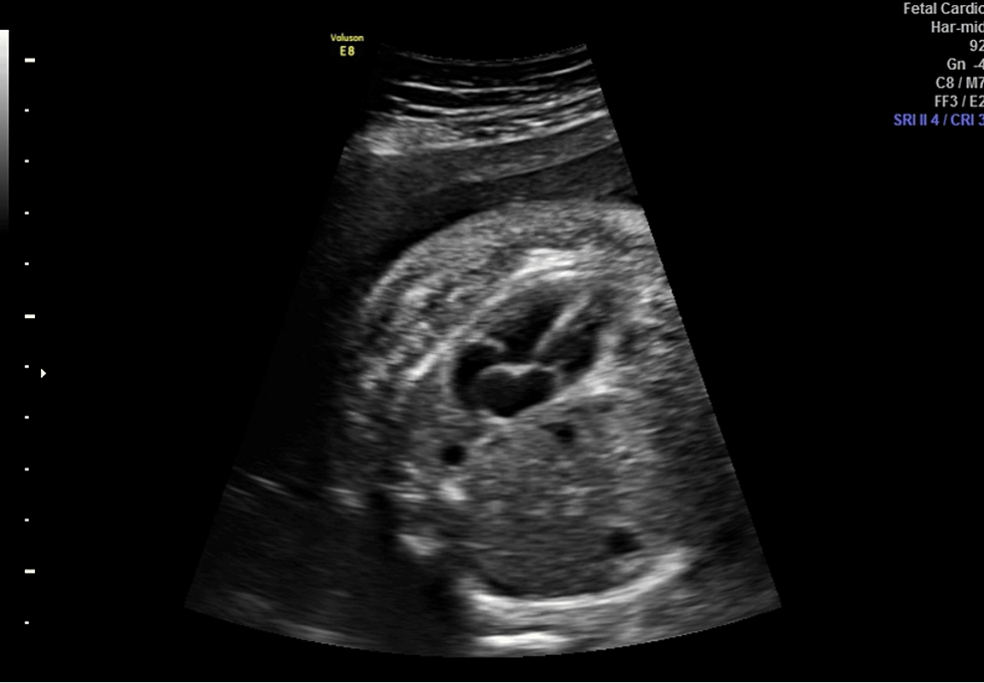
One week after surgery, no pleural effacement was left

In the first week, the fetus presented an evident reduction of pleural effusion (-71.4%), ascites (-94.1%), and subcutaneous edema (-40%), while CCO remained stable. Two weeks later, no edema, ascites, or pleural effusion was present (Figure 2).

**Figure 3:**
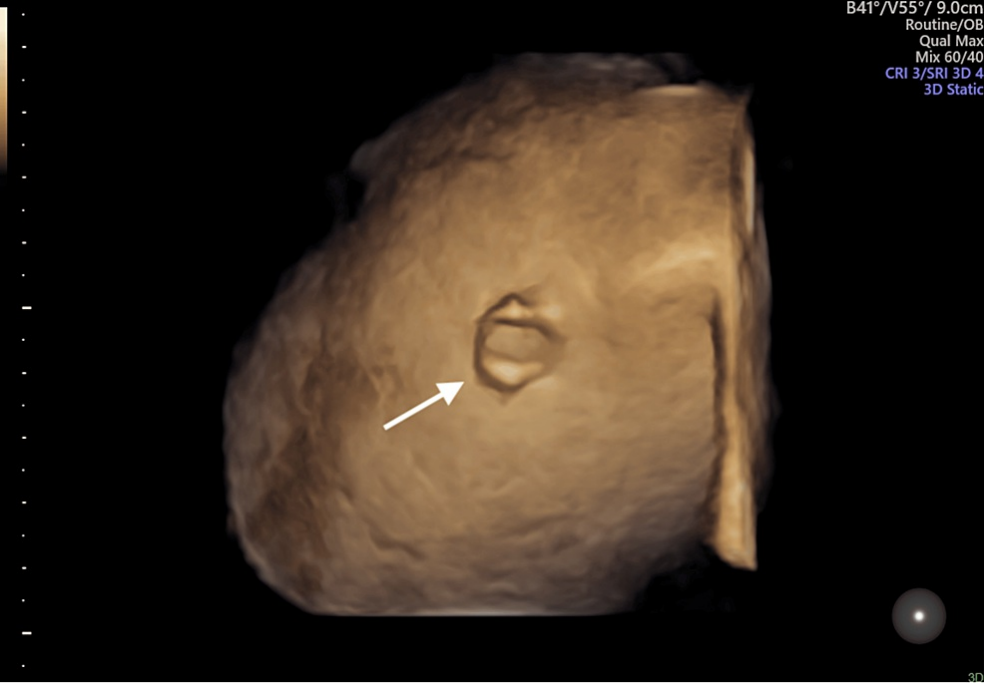
3D surface rendering confirms the correct thoracoamniotic shunt position in the right hemithorax (arrow)

**Figure 4:**
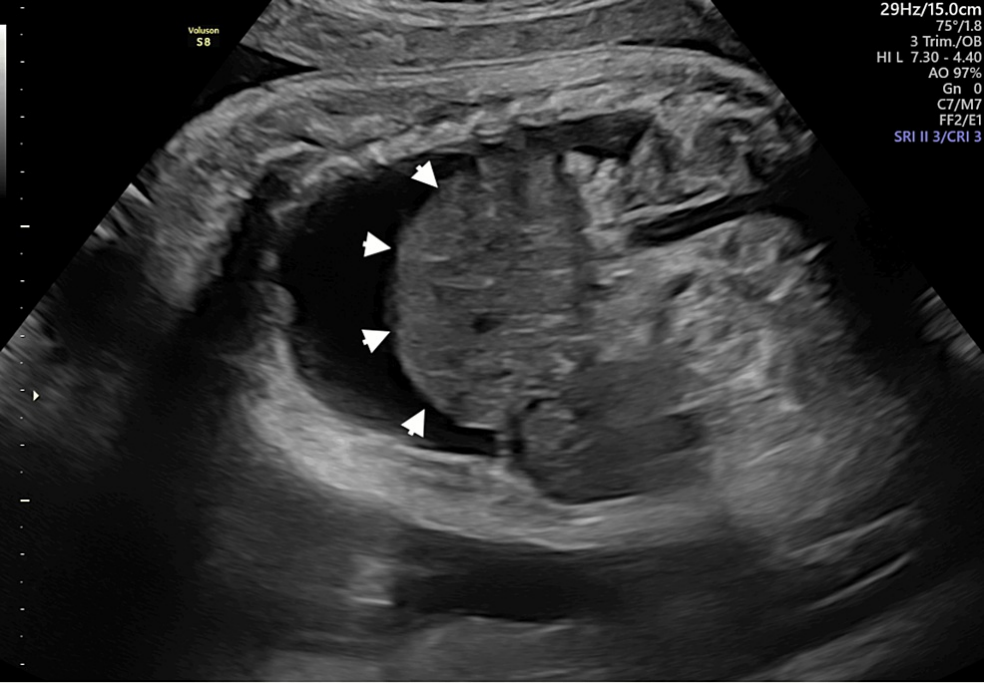
Sagittal scan reveals a “mushroom-like” liver herniation in the mediastinal cavity with hydrothorax (arrowheads)

Although the liver occupied most of the right hemithorax, CCO increased significantly, reaching a Z-score of -0.2, as well as right and left cardiac output (Z-scores: -0.3 and -0.8, respectively), with a slight reduction of mediastinal shift. Three weeks after the procedure, despite the correct position of the shunt confirmed by 3D volume acquisition (Figure 3), slight ascites with right pleural effusion and bulging of the liver in the homolateral hemithorax relapsed (Figure 4), along with a worsening of the cardiac performance (CCO Z-score: -1.4), suggesting a possible shunt occlusion.

The day after, at 33 weeks of gestation, the patient was hospitalized due to uterine contractions, and the same day, an emergency C-section was performed for precipitous labor in breech presentation. A male newborn of 1870 grams was delivered, and the shunt was promptly removed. At birth, the neonate showed a defective adaptation (APGAR score of 5-7-8 at one, five, and 10 minutes, respectively) with a low heart rate (<100 bpm) and tachypnea. Cord blood analysis showed moderate acidosis (pH 7.09). The neonate was immediately intubated (a fraction of inspired oxygen was 100%) with prompt normalization of cardiac frequency. Inhaled nitric oxide was started at three hours of life (40 ppm), and two doses of surfactant (100 mg/kg) were administered in the subsequent six hours. Echocardiography confirmed the mediastinal shift to the left, with the liver completely herniated in the right hemithorax and severe left lung hypoplasia. No right lung parenchyma was observed. Due to severe pulmonary hypertension suggestive of congestive cardiac failure, continuous intravenous administration of vasopressin (12 UI, 0.5 mL/h) and milrinone (0.75 mcg/kg/min) was started. Cerebral ultrasound showed a vasoconstriction in the anterior cerebral artery with flow reversed. Cardiorespiratory conditions progressively worsened, and at 12 hours of life, the neonate died despite intensive resuscitation with cardiac massage and noradrenaline 1.5 mg (0.1 mL/h) and epinephrine 1 mg (0.1 mL/h) administration. At the autopsy, the right hemidiaphragm was present as a thin serous membrane, suggestive of diaphragmatic eventration with muscular agenesis of its sternal, costal, and lumbar portions. This led to the herniation of the liver and, partially, of the right adrenal gland into the thorax. The left lung showed hypoplasia and atelectasis. The right lung was present as an underdeveloped, thin structure. The amniocentesis performed during the shunt placement revealed a normal karyotype and a negative CGH array. The patient provided written consent for the publication of anonymous clinical data regarding her and her son.

## Discussion

RCDH is associated with a relevant neonatal mortality rate (between 30% and 45%), which is higher than isolated left-sided defects [6]. The postnatal outcome is expected to worsen significantly in cases of association with chromosomal or genetic abnormalities or other defects [7]. The effectiveness of fetal endoscopic tracheal occlusion (FETO) in right-sided defects has been reported to be lower than in left-sided cases [8]. Despite this evidence, recent studies are encouraging us to extend the indication to FETO intervention in the event of RCDH [9]. In cases complicated by NIH, the appropriate management is uncertain. The present report provides important information about the beneficial cardiovascular effects of a thoracoamniotic shunt in the event of NIH with voluminous hydrothorax complicating RCDH. The exact pathogenesis of cardiogenic NIH is still not exhaustively understood [10]. The compression due to liver herniation in the right hemithorax causes an evident mediastinum shift and severe right lung hypoplasia. The cardiac malposition obstructs an adequate venous return from the inferior vena cava, reducing the preload and, consequently, the CCO resulting from both ventricles. Congestive heart failure, characterized by increasing ventricular end-diastolic pressure, implies higher central venous pressure. This leads to a reduction in systemic lymphatic return and, consequently, an increased movement of fluid to the interstitial space, culminating in NIH [11]. Several ultrasound parameters have been described to assess fetal cardiac function [12]. Cardiac output, defined as the volume of blood pumped by each ventricle per minute, is a reliable and reproducible method to evaluate systolic function [13]. Biventricular CCO, resulting from the sum of each ventricle's cardiac output, has been described as a trustworthy measure of fetal myocardial function, even in cases of cardiac adaptation to extracardiac anomalies [14].

## Conclusions

In this report, we describe the longitudinal trend of fetal cardiac function after the placement of a thoracoamniotic shunt. Very interestingly, the improvement in cardiac output was directly related to the reduction of the hydrops. The strict correlation between lymphatic effusions in multiple body compartments and CCO has never been described elsewhere, as have the longitudinal evaluations of such parameters in fetuses subjected to in-utero treatments. This reproducible ultrasound parameter could be used to quantify fetal heart function’s modification in cases of extracardiac anomalies, providing information about the efficacy of in-utero treatments and the clinical trend of cardiac adaptation. Due to the high mortality rate of RCDH complicated by NIH and its poorer outcomes compared to left-sided defects, thoracoamniotic shunts cannot be considered, to date, a safe and efficient attempt to improve fetal and neonatal survival rates. Nevertheless, further studies are unequivocally helpful in providing more information about the CCO cut-off and its trend to better understand fetal circulation pathophysiology and the possible cardiac improvements in terms of morbidity and mortality for fetuses affected by cardiac impairment.
